# Genetics of Nonsyndromic Congenital Hearing Loss

**DOI:** 10.1155/2016/7576064

**Published:** 2016-02-18

**Authors:** Oguz Kadir Egilmez, M. Tayyar Kalcioglu

**Affiliations:** Department of Otorhinolaryngology, Faculty of Medicine, Istanbul Medeniyet University, 34722 Istanbul, Turkey

## Abstract

Congenital hearing impairment affects nearly 1 in every 1000 live births and is the most frequent birth defect in developed societies. Hereditary types of hearing loss account for more than 50% of all congenital sensorineural hearing loss cases and are caused by genetic mutations. HL can be either nonsyndromic, which is restricted to the inner ear, or syndromic, a part of multiple anomalies affecting the body. Nonsyndromic HL can be categorised by mode of inheritance, such as autosomal dominant (called DFNA), autosomal recessive (DFNB), mitochondrial, and X-linked (DFN). To date, 125 deafness loci have been reported in the literature: 58 DFNA loci, 63 DFNB loci, and 4 X-linked loci. Mutations in genes that control the adhesion of hair cells, intracellular transport, neurotransmitter release, ionic hemeostasis, and cytoskeleton of hair cells can lead to malfunctions of the cochlea and inner ear. In recent years, with the increase in studies about genes involved in congenital hearing loss, genetic counselling and treatment options have emerged and increased in availability. This paper presents an overview of the currently known genes associated with nonsyndromic congenital hearing loss and mutations in the inner ear.

## 1. Introduction

Hearing loss (HL) is a common disorder, and congenital hearing impairment affects nearly 1 in every 1000 live births; it is one of the most distressing disorders and the most frequent birth defect in developed societies [1]. Hearing impairment affects speech development, language acquisition, and education in children and, as a result, often leads to decreased opportunities in work life as those with hearing loss move to isolating themselves from society. In the US, it is estimated that the social costs of untreated hearing loss over the course of a lifetime can reach up to $1.1 million for every untreated person [2]. These costs could be decreased by 75 percent with early intervention and treatment [3].

Hereditary hearing loss accounts for almost 50% of all congenital sensorineural hearing loss cases, and it is caused by genetic mutations [4]. Deafness can be the result of a mutation in a single gene or a combination of mutations of different genes; it can also be a result of environmental causes such as trauma, medications, medical problems, and environmental exposure or the result of an association between environmental factors and genetics [5].

HL can be either nonsyndromic, which is restricted to the inner ear, or syndromic, a part of multiple anomalies affecting the body. Nonsyndromic HL can further be categorised by its mode of inheritance. Approximately 20% of nonsyndromic sensorineural hearing loss (NSSHL) is inherited as autosomal dominant, which is also referred to as DFNA; this type of hearing loss is usually delayed onset. Eighty percent of inherited HL is autosomal recessive (DFNB), in which hearing loss is generally congenital, but some forms may emerge later in life. The inheritance of the remaining types of HL is either mitochondrial or X-linked (DFN) (less than 1 percent) [2]. To date, 125 deafness loci have been reported in the literature: 58 DFNA loci, 63 DFNB loci, and 4 X-linked loci (http://hereditaryhearingloss.org/) [6].

Many genes are involved in inner-ear function, and the ear is very sensitive to mutations in genetic loci. This is because the physiology and structure of the inner ear are unique and unlike other anatomical locations. Mutations in genes that control the adhesion of hair cells, intracellular transport, neurotransmitter release, ionic hemeostasis, and cytoskeletons of hair cells can lead to malfunctions of the cochlea and inner ear.

In recent years, with the increase in studies of genes involved in congenital hearing loss, genetic counselling and treatment options have emerged and increased in availability. In diagnostic tests, genes that are common causes of hearing loss, such as* GJB2*,* GJB6*,* SLC26A4*, and* OTOF*, are frequently involved [7]. The results of these tests can be used when counselling parents about the prognosis of a child's hearing loss, predicting recurrence in the future offspring and taking into consideration therapeutic options like cochlear implantation [2]. In recent studies, some viral vectors were delivered into the inner ear to replace the normal copy of the gene with the defective gene causing hearing loss. In an animal study, an adenovirus-delivered* SLC17A8* (VGLUT-3; vesicular glutamate transporter 3) was found to restore hearing in the mice. In another study, hair cell development and regeneration were induced by delivering the* ATOH1* gene [8, 9].

This minireview has presented an overview and described the currently known genes associated with nonsyndromic congenital hearing loss and mutations that cause malfunctional proteins in the inner ear (Table 1).

## 2. Genes and Proteins Related to Nonsyndromic Hearing Loss

### 2.1. Adhesion Proteins

The stereocilia of hair cells in the cochlea are linked and interconnected to the tectorial membrane by different adhesion proteins. Hair bundles are stabilized by a set of temporary links such as transient lateral links and ankle links. These links also induce growth and maturation with signalling complexes [10]. In mature hair cells, stereocilia are connected by tectorial attachment crowns, horizontal top connectors, and tip links [2]. To date, several genes related to the linking apparatus have been reported. These are DFNA4 (*CEACAM16* (carcinogenic antigen-related cell adhesion molecule 16)) [11], DFNB12 (*CDH23* (cadherin 23)) [12], DFNB16 (*STRC* (stereocilin)) [13], DFNB18 (*USH1C* (harmonin)) [14, 15], DFNB22 (*OTOA* (otoancorin)) [16], DFNB23 (*PCDH15* (protocadherin 15)) [17], DFNB31 (*WHRN* (whirlin)) [18], DFNB66/67 (*TMHS* (tetraspan membrane protein)) [19], and DFNB84 (*PTPRQ* (tyrosine phospate receptor Q)) [20].

The* PTPRQ* and* TMHS* genes, as well as cadherin 23 and protocadherin 15, are parts of the transient lateral link. During development, they prevent the fusion of each stereocilium themselves [2]. In mature hair cells, they become the main parts of the tip link and act as a gate, channelling mechanotransduction and providing stability, taking a central role in auditory function [21].

Whirlin and harmonin regulate the link complexes and serve as scaffolding proteins. Mutations in these proteins cause autosomal recessive type hearing loss, but Sans, which is a third scaffolding protein, is related to a complex syndromic hearing loss, Usher syndrome. The other genes,* USH2α* and* VLGR1b*, are also associated with Usher syndrome, and they are part of the stereocilial ankle link [22].

Stereocilin is an extracellular matrix protein that attaches the tallest stereocilia of the outer hair cells to the tectorial membrane [13]. The attachment site of this tectorial membrane is generally formed by* CEACAM16*. In a similar way, otoancrin also attaches nonsensory cells to the tectorial membrane [16].

### 2.2. Transport Proteins

In the inner ear, all parts of the myosin family can be used for the transportation of different proteins. When using ATP, these myosin proteins bind to the actin cytockeleton and move forward. Binding sites for carried proteins are on the carboxyl-terminal tails of the transport proteins [23]. The myosins related to hereditary hearing loss are myosin Ia (DFNA48) [24], myosin IIIa (DFNB30) [25], myosin VI (DFNA22/DFNB37) [26, 27], myosin VIIa (DFNA11/DFNB2) [28, 29], nonmuscle myosin heavy chain IX (DFNA17) [30], nonmuscle myosin heavy chain XIV (DFNA4) [31], and myosin XVa (DFNB3) [32]. They all have their own unique functions in the inner-ear hair cells [2].

### 2.3. Proteins of Synapses

VGLUT3, which is a vesicular glutamate receptor, plays a role in the inner hair cells' synapses. It is encoded by* SLC17A8* in the DFNA25 locus and related to autosomal recessive hearing loss [33]. This protein regulates both the exocytosis and the endocytosis of glutamate. Otoferlin (encoded by* OTOF*) is a protein that works with myosin VI at the synaptic cleft of the inner hair cell and plays a role in the calcium-dependent fusion of vesicles to the plasma membrane. As a result, glutamate is released and the afferent neuron is excited [34]. In an animal study with* OTOF* and* SLC17A8* knockout mice, there was a reduction in the number of postsynaptic ganglion cells, and it was concluded that these proteins are very important for the preservation and development of normal hearing [35].

### 2.4. Electromotility

The cochlea is sensitive and selective to sounds delivered by the outer hair cells. This is introduced with a process called electromotility, and a protein called Prestin is thought to be responsible for this [2]. It changes the membrane's potential and enables the outer hair cell length to be altered. When this occurs, the outer hair cell becomes longer upon hyperpolarization and shorter upon depolarization, so it amplifies its sensitivity to the sound [36]. This protein is encoded as* SLC26A5* and was first described by Zheng et al. in 2000 [37]. Mutations in* SLC26A5* are the cause of DFNB61 hearing loss [38].

### 2.5. Cytoskeleton

Mutations in some genes associated with the organisation of the cytoskeleton can cause NSSHL; these are* ESPN* (espin),* RDX* (radixin),* TRIOBP* (trio-binding protein),* ACTG1* (*γ*-actin),* TPRN* (taperin),* DIAPH1* (diaphanous), and* SMPX* (small muscle protein, X-linked).

The protein espin provides stability to the stereocilial cytoskeleton. A mutation in* ESPN* can cause DFNB36 and autosomal dominant hearing loss [39]. More steriocillia stability can be achieved with radixin. It links actin filaments to the plasma membrane and presents along the stereocilia. Mutations in* RDX* can cause DFNB24 and autosomal recessive deafness [40]. *γ*-actin acts as a building block for the stereocilia of hair cells. These stereocilia are constantly undergoing depolymerisation at the base and actin polymerisation at the tip [41]. Mutations in* ACTG1* can cause DFNA20/26 and autosomal dominant hearing loss [42, 43]. Via a constant remodelling process, other proteins are also important for continuity. Diaphanous 1 regulates the reorganisation and polymerisation of actin monomers into polymers. It is encoded as* DIAPH1*, and mutations in this gene can cause DFNA1 and autosomal dominant hearing loss [44]. The binding and organisation of *γ*-actin at the base of stereocilia are provided by two isoforms of the* TRIOBP* gene. Mutations in isoforms that are* TRIOBP4* and* TRIOBP5* can cause DFNB28 and autosomal recessive type hearing loss [45, 46]. Another protein, taperin, is localised in the base of the stereocilia and associated with DFNB79 [47]. Small muscle protein X-linked, encoded as* SMPX* (DFN4), has a function in stereocilial development and maintenance in response to the mechanical stress to which stereocilia are subjected [48].

### 2.6. Ion Homeostasis and Gap Junctions

The cochlea has two types of fluids: perilymph, which is high in sodium and low in potassium, and endolymph, which is high in potassium and low in sodium; this condition makes a highly positive potential (+80 mV) called endocochlear potential. Potassium influx into the hair cells causes depolarisation and, after that, the hair cell repolarises and moves cations back into the endolymph. This ion homeostasis involves tight junction protein 2 (*TJP2*), tricellulin (*MARVELD2*/*TRIC*), claudin 14 (CLDN14), KCNQ4 (*KCNQ4*), Barttin (*BSND*), ATP2b2 (*ATP2b2*/*PMCA2*), some connexins (*GJBs*), and pendrin (*SLC26A4*), and they are all related to hereditary hearing loss [2].

In a mutation of* CLDN14* in DFNB29, claudin 14 protein will be absent or dysfunctional, and the space of Nuel that surrounds the basolateral surface of outer hair cells is affected and might change its electrical potential [49]. Similarly, tricellulin, which is encoded as* MARVELD2*/*TRIC*, causes DFNB49 when mutated, and it is functioning as tight junction that connects the cells together [45]. Tight junction protein 2, encoded as the* TJP2* gene, binds tight junctions to the actin cytoskeleton, and mutations cause DFNA51 and autosomal dominant type hearing loss [50].

*KCNQ4* encodes a protein forming a voltage-gated potassium channel. It is expressed in outer hair cells and, if mutated, causes an autosomal dominant type HL, DFNA2a [51]. It aids in the repolarisation of outer hair cells and regulates the sensitivity to sound.

Barttin and pendrin, encoded as* BSND* and* SLC26A4*, respectively, are involved in both nonsyndromic and syndromic HL. Pendrin is an anion exchanger and plays a crucial role in the acid-base balance. Both syndromic (Pendred's syndrome, associated with goiter) and nonsyndromic HL (DFNB4) are related to the extent of the mutation in* SLC26A4* [52]. Barttin protein is one of the subunits of the chloride channel. Mostly, mutations in* BSND* can cause Bartter syndrome, associated with hearing loss and renal abnormalities, but DFNB73 has also been attributed to a mutation in* BSND* and causing nonsyndromic deafness [53].

A gap junction is a channel extending over two adjacent membranes that enables the exchange of various molecules and ions in the cochlea. These junctions are made up of proteins called connexins. These junctions also play a role in the recycling of potassium ions needed for normal hearing. it is the most common cause of nonsyndromic HL and was the first identified gene is* GJB2;* it is encoded as connexin 26 (DFNA3a/DFNB1a) [54]. Other connexins related to nonsyndromic HL are connexin 30 (*GJB6*, DFNA3b/DFNB1b) [55, 56] and connexin 31 (*GJB3*, DFNA2b/DFNB91) [57, 58].

### 2.7. Others

There are also extracellular matrix proteins, that is,* TECTA* (*α*-tectorin),* COL11A2* (type XI collagen *α*2), and* COCH* (cochlin), and transcription factors, such as* POU4f3* (class 4 POU),* POU3f4* (class 3 POU),* MIR96* (microRNA 96),* GRHL2* (grainy-head-like 2),* ESRRB* (oestrogen-related receptor *β*), and* EYA4* (eyes absent 4) involved in hereditary HL.

## 3. Conclusion

This review presents an overview and description of the currently known genes related to hereditary NSSHL. The functions of these genes will be better understood with time, and more genes leading to hearing loss will be discovered soon. With new studies and continued examination, the function of the cochlea will be better understood, and novel molecular and gene therapies for human sensorineural HL will hopefully be developed.

## Figures and Tables

**Table 1:** Genes related with nonsyndromic hearing loss.

Locus	Gene	Chromosomal localization	Type of inheritance	Protein	Function	Reference
DFNA1	DIAPH1	5q31	AD	Diaphanous 1	Actin polymerisation (cytoskeleton)	[44]
DFNA2A	KCNQ4	1p34	AD	KCNQ4	Voltage-gated K^+^ channel (ion haemostasis)	[51]
DFNA2B	GJB3 (Cx31)	1p34	AD	Connexin 31	Gap junction (ion haemostasis)	[57]
DFNA3A	GJB2 (Cx26)	13q12	AD	Connexin 26	Gap junction (ion haemostasis)	[54]
DFNA3B	GJB6 (Cx30)	13q12	AD	Connexin 30	Gap junction (ion haemostasis)	[55]
DFNA4	MYH14	19q13	AD	Nonmuscle myosin heavy chain XIV	Transport	[31]
DFNA4	CAECAM16	19q13	AD	Carcinogenic antigen-related cell adhesion molecule 16	TM attachment crown (adhesion)	[11]
DFNA8/12	TECTA	11q22-24	AD	A-tectorin	Stability and structure of TM (ECM)	[28]
DFNA9	COCH	14q12-q13	AD	Cochlin	Structure of spiral limbus	[30]
DFNA10	EYA4	6q22-q23	AD	Eyes absent 4	Regulation of transcription (transcription factor)	[42]
DFNA11	MYO7A	11q12.3-q21	AD	Myosin VIIa	Transport	[43]
DFNA13	COLL11A2	6p21	AD	Type XI collagen *α*2	Stability and structure of TM (ECM)	[26]
DFNA15	POU3F4	5q31	AD	Class 3 POU	Regulation of transcription (transcription factor)	[33]
DFNA17	MYH9	22q	AD	Nonmuscle myosin heavy chain IX	Transport	[24]
DFNA20/26	ACTG1	17q25	AD	*γ*-actin	Building cytoskeleton (cytoskeleton)	[50, 56]
DFNA22	MYO6	6q13	AD	Myosin VI	Regulation of exocytosis, anchoring stereocilia (cytoskeleton)	[29]
DFNA25	SLC17A8	12q21-24	AD	VGLUT-3	Regulation of exocytosis and endocytosis of glutamate (transport)	[32]
DFNA28	TFCP2L3	8q22	AD	Transcription factor CP2-like 3	Regulation of transcription (transcription factor)	[52]
DFNA48	MYO1A	12q13-q14	AD	Myosin Ia	Transport	[34]
DFNA50	MIR96	7q32	AD	MicroRNA96	Regulation of transcription (transcription factor)	[12]
DFNA51	TJP2		AD	Tight junction protein 2	Cell cycle signaling, binding tight junctions to membrane	[13]
DFNA56	TNC	9q31.3-q34.3	AD	Tenascin-C	Stability and structure of TM (ECM)	[14]
DFNA64	SMAC/DIABLO	12q24.31-12q24.32	AD	Second Mitochondria-Derived Activator of Caspase/Direct Inhibitor of Apoptosis protein Binding protein with a low pI	Cell cycle signaling	[15]
DFNA65	TBC1D24	16p13.3	AD	Tbc1 domain family, member 24	Encoding a GTPase-activating protein expressed in the cochlea	[16, 17]
DFNA67	OSBPL2	20q13.2-q13.33	AD	Oxysterol-binding Protein-like Protein 2	Intracellular transport of lipids, particularly oxysterol (transport)	[40, 45]
	HOMER2	15q25.2	AD	Homer, drosophila, homolog of 2	Negative regulator of T cell activation	[46]
DFNB1A	GJB2 (Cx26)	13q11-q12	AR	Connexin 26	Gap junction (ion haemostasis)	[13]
DFNB1B	GJB6 (Cx30)	13q12	AR	Connexin 30	Gap junction (ion haemostasis)	[49]
DFNB2	MYO7A	11q	AR	Myosin VIIa	Transport	[25, 43]
DFNB3	MYO15A	17p11.2	AR	Myosin Xva	Transport	[18]
DFNB4	SLC26A4	7q31	AR	Pendrin	Acid-base balance of endolymph (ion haemostasis)	[39]
DFNB9	OTOF	2p23-p22	AR	Otoferlin	Fusion of synaptic vesicles with Ca^+2^ (transport)	[38]
DFNB12	CDH23	10q21-q22	AR	Cadherin 23	Lateral and tip link (adhesion)	[19]
Modifier of DNFB12	ATP2b2/PMCA2	1p32.3	AR	ATP2b2	ATP dependent Ca^+2^ pump	[53]
DFNB16	STRC	15q15	AR	Stereocilin	TM attachment links (adhesion)	[20]
DFNB18	USH1C	11p15.1	AR	Harmonin	Scaffolding protein (adhesion)	[48, 58]
DFNB21	TECTA	11q22-q24	AR	*α*-tectorin	Stability and structure of TM (ECM)	[10]
DFNB22	OTOA	16p12.2	AR	Otoancorin	TM attachment to nonsensroy cells (adhesion)	[21]
DFNB23	PCDH15	10q21-q22	AR	Protocadherin 15	Lateral and tip link (adhesion)	[22]
DFNB24	RDX	11q23	AR	Radixin	Actin binding to plasma membrane (cytoskeleton)	[23]
DFNB28	TRIOBP	22q13.1	AR	Trio-binding protein	Actin binding and organisation (cytoskeleton)	[27, 35]
DFNB29	CLDN14	21q22.3	AR	Claudin 14	Tight junction (ion haemostasis)	[36]
DFNB30	MYO3A	10p11.1	AR	Myosin IIIa	Transport	[37]
DFNB31	WHRN	9q32-q34	AR	Whirlin	Scaffolding protein (adhesion)	[41]
DFNB35	ESRRB	14q21.1-q24.3	AR	Oestrogen-related receptor *β*	Regulation of transcription (transcription factor)	[47]
DFNB36	ESPN	1p36.3-p36.1	AR	Espin	Actin crosslinking and bundling (cytoskeleton)	[59]
DFNB37	MYO6	6q13	AR	Myosin VI	Regulation of exocytosis, stereocilia anchoring (transport)	[60]
DFNB49	TRIC	5q12.3-q14.1	AR	Tricellulin	Tight junction (ion haemostasis)	[53]
DFNB53	COL11A2	6p21.3	AR	Type XI collagen *α*2	Stability and structure of TM (ECM)	[61]
DFNB61	SLC26A5		AR	Prestin	Electromotility	[62]
DFNB67	TMHS	6p21.3	AR	Tetraspan membrane protein	Transient link (adhesion)	[63]
DFNB73	BSND		AR	Barttin	K^+^ channel maturation and trafficking (ion haemostasis)	[64]
DFNB79	TPRN	9q34.3	AR	Taperin	Actin regulation (cytoskeleton)	[65]
DFNB84	PTPRQ	12q21.31-q21.2	AR	Protein tyrosine phosphate receptor Q	Transient link (adhesion)	[66]
DFNB91	GJB3	6p25	AR	Connexin 31	Gap junction (ion haemostasis)	[67]
DFNB93	CABP2	11q13.2	AR	Calcium-binding protein 2	(ion haemostasis)	[68]
DFNB94	NARS2	11q14.1	AR	Asparaginyl-tRNA synthetase 2	(transport)	[69]
DFNB97	MET	7q31.2	AR	MET protooncogene	Cell-surface receptor for hepatocyte growth factor (adhesion)	[70]
DFNB98	TSPEAR	21q22.3	AR	Thrombospondin-type laminin g domain and ear repeats	Cell permeabilization (transport)	[71]
DFNB99	TMEM132E	17q12	AR	Transmembrane protein 132e	Extracellular receptor	[72]
DFNB101	GRXCR2	5q32	AR	Glutaredoxin, cysteine-rich 2	Organisation of stereocilia (adhesion)	[73]
DFNB102	EPS8	12p12.3	AR	Epidermal growth factor receptor pathway substrate 8	Regulating Rac-specific GEF activity (transcription factor)	[74]
DFNB103	CLIC5	6p21.1	AR	Chloride intracellular channel 5	(ion haemostasis)	[75]
	FAM65B	6p22.3	AR	Family with sequence similarity 65, member b	(Cytoskleton)	[76]
Usher syndrome	SANS/USH1G	17q24-25	AR	SANS	Scaffolding protein (adhesion)	[77]
Usher syndrome	USH2A	1q41	AR	Usherin	Ankle link (adhesion)	[78]
Usher syndrome	VLGR1B		AR	Very large G protein-coupled receptor 1	Ankle link (adhesion)	[79]
DFN2	PRPS1	Xq22.3	X-linked	Phosphoribosylpyrophosphate synthetase 1	Purine and pyrimidine biosynthesis	[80]
DFN3	POU3F4	Xq21	X-linked	Class 3 POU	Regulation of transcription (transcription factor)	[81]
DFN6	SMPX	Xp21.2	X-linked	Small muscle protein X-linked	Stereocilial development and maintenance (cytoskeleton)	[82]
DFNX6	COL4A6	Xq22.3	X-linked	Collagen, type IV, alpha-6	Stability and structure of TM (ECM)	[83]

DFNA = nonsyndromic deafness, autosomal dominant; DFNB = nonsyndromic deafness, autosomal recessive; AD = autosomal dominant; AR = autosomal recessive; TM = tectorial membrane; ECM = extracellular matrix; Ca^+2^ = calcium ion; K^+^ = potassium ion.
